# Electrochemical Method for the Design of New Possible Gadolinium-Based Contrast Agents

**DOI:** 10.3390/nano14241979

**Published:** 2024-12-10

**Authors:** Claudia Carbone, Aaron Stoeckle, Manuel Minardi, Fulvio Uggeri, Luciano Lattuada, Alessandro Minguzzi, Alberto Vertova

**Affiliations:** 1 Laboratory of Applied Electrochemistry, Dipartimento di Chimica, Università degli Studi di Milano, Via Golgi, 19, 20133 Milan, Italy; claudia.carbone@unimi.it (C.C.); manuel.minardi2@gmail.com (M.M.); alberto.vertova@unimi.it (A.V.); 2 Institute of Physical Chemistry and Electrochemistry, Leibniz University Hanover, Callinstr. 3A, 30167 Hanover, Germany; aaron.stoeckle@web.de; 3 Bracco Imaging SpA, via Egidio Folli 50, 20134 Milano, Italy; fulvio.uggeri@bracco.com (F.U.); luciano.lattuada@bracco.com (L.L.)

**Keywords:** gadolinium-based contrast agents, ZnHCF nanoparticles, gadolinium ion

## Abstract

Magnetic resonance imaging (MRI) is a technique that employs strong magnetic fields and radio frequencies to generate detailed images of the body’s interior. In oncology patients, gadolinium-based contrast agents (GBCAs) are frequently administered to enhance the visualization of tumors. Those contrast agents are gadolinium chelates, characterized by high stability that prevents the release of the toxic gadolinium ion into the body. This work is part of the research for alternative nanoscaled GBCAs. Following the synthesis and characterization of zinc hexacyanoferrate nanoparticles, gadolinium ions were successfully incorporated into a hexacyanoferrate-based matrix, deposited on FTO-coated glass used as working electrode in a gadolinium salt solution, by applying a fixed potential determined through cyclic voltammetry studies. The presence of gadolinium inside the matrix was confirmed by EDX.

## 1. Introduction

Magnetic resonance imaging (MRI) is a widely used diagnostic technique for the detection and staging of various types of cancer. This method exploits the differences in spin relaxation times (primarily T_1_ = spin-lattice relaxation time) of water protons in different biological tissues, when exposed to strong magnetic fields and radio frequency irradiation [1,2].

The intravenous administration of solutions of gadolinium-based contrast agents (GBCAs) is commonly employed to increase the water relaxation time rate (R_1_ = 1/T_1_) of water protons (phenomenon known as paramagnetic relaxation enhancement (PRE)), resulting in an improved tumor to background ratio. The relaxivity is the primary parameter that characterizes the sensitivity and effectiveness of MRI contrast agents, reflecting variations in relaxation rates of water protons as a function of the concentration [3,4]. MRI with contrast exploits the hypervascularization of tumors, due to neoangiogenesis: in these areas, the contrast agent may extravasate, thereby highlighting pathological lesions [5].

Specifically, Gd^3+^, a paramagnetic ion with seven symmetric unpaired electrons, accelerates the T_1_ of nearby water protons: in the medical imaging technique, variations in relaxation rates are translated into grayscale in MRI, leading to a clearer visualization of the disease due to enhanced contrast [6,7].

Currently, GBCAs are used clinically for the enhancement of the contrast through acceleration of T_1_. These consist of Gd^3+^ linear (DTPA) or macrocyclic (DOTA, HP-DO3A) complexes with nine coordination sites. The complexes feature an octadentate polyaminocarboxylate chelator on eight positions and an inner-sphere water molecule on the ninth site [8]. This water molecule rapidly exchanges with bulk water, transferring the PRE effect to the surrounding area, thus enhancing the relaxivity of nearby protons and the signal intensity. The octadentate ligand provides stability to the complex, preventing the release of Gd^3+^, which is toxic for the human body [9], while leaving one position available for water coordination.

Recently, various new types of MRI contrast media have been developed, many of them based on nanoparticles [10,11]. Bioresponsive GBCAs are particularly promising; they are designed to be conditionally retained or activated in vivo in response to specific biochemical events of interest. Consequently, any observed changes in MR signal can serve as a read-out for molecular events [1]. In addition, gadolinium-based nanoscale MRI contrast agents have been widely studied for tumor detection in medical diagnoses [12]. For instance, gadolinium chelates have been incorporated into nanocarriers to enhance the density of gadolinium ions [13]. Conversely, Gd^3+^-containing nanoscale MRI contrast agents have been developed, offering the advantage of a high payload of gadolinium ion within individual nanoparticles [14]. Inorganic nanoparticles incorporating on other paramagnetic ions such as Mn^2+^ or Fe^3+^ have also been investigated; however, the focus remains on gadolinium-based systems due to the high number of unpaired electrons that contribute to the enhancement of water proton relaxivity in diseased tissues [13,14]. Notably, nanodiamonds have emerged as a promising base for the development of innovative MRI contrast agents with various functionalized systems demonstrating remarkable performance. Examples include polyvinylpyrrolidone (PVP)-coated Gd-grafted detonation nanodiamonds (DND), offering significantly higher signal intensity than traditional Gd-DOTA [15]; Gd(III)–nanodiamond conjugates with one of the highest reported per-Gd(III) relaxivities [16]; and Gd-DTPA-modified nanodiamonds, which achieve excellent dispersion in biological media and show high T_1_-weighted signal intensity for advanced vascular and lymphatic imaging [17]. Additionally, manganese-conjugated nanodiamonds have proven to enhance both T_1_- and T_2_-weighted MR images [18].

The concept behind this project is inspired by research on Zn-ion batteries and their intercalation chemistry. Recently, Prussian blue analogue materials (PBAs), which resemble Prussian blue in structure but consist of different cations, have gained significant interest as active materials for cathodes of metal-ion batteries [19,20,21,22,23,24]. These hexacyanoferrate-bridged structures are characterized by easy and cost-effective synthesis processes, high ionic conductivity within the crystal lattice, and excellent safety and environmental compatibility. More importantly, they exhibit a high capacity for the reversible electrochemical insertion of various metal ions with minimal volume change, making them suitable for a wide range of applications, also in nanoheterostructures [19,25].

Although they are promising candidates for aqueous rechargeable metal-ion batteries, mechanistic studies about the intercalation remain limited. Progress has been made by Honhyan Li et al., who investigated the intercalation mechanism of Na^+^ and K^+^ in Berlin green using *operando* X-ray diffraction and Raman spectroscopy [26].

Beyond their application in energy storage systems, Prussian blue nanoparticles (PBNPs) and their analogues have attracted considerable attention in the medical sector for their biocompatibility, good thermal stability, and unique properties. They hold potential for applications in drug delivery, therapeutic agents, immunosensors, and imaging [27,28]. For example, they have been explored for the treatment of Alzheimer’s disease to alleviate cognitive decline [29] and for addressing atherosclerosis to mitigate inflammation by scavenging reactive oxygen species (ROS) [30]. This property is particularly important for medical applications as ROS are implicated in apoptosis and cell death in various diseases [31]. Furthermore, Prussian blue analogues have excellent antibacterial activity [32].

As mentioned earlier, these materials have been investigated also to enhance medical imaging quality, including in photoacoustic, multimodal imaging, and MRI [27]. Notably, there are instances of biocompatible nanoparticles of gadolinium-incorporated Prussian blue (or analogues) that can act as effective MRI contrast agents by enhancing the longitudinal relaxivity of water protons [33,34,35].

The present research aimed to develop a new electrochemical method to create Gd^3+^-rich nanoparticles of zinc hexacyanoferrate, with the chemical formula Zn_3_[Fe(CN)_6_]_2_, hereafter referred to as ZnHCF throughout the text, a type of Prussian blue analogue containing zinc, which could serve as high-efficiency GBCAs. Electrochemistry can facilitate the incorporation of cations into the structure of the PBA through the application of a voltage. By controlling the applied potential and duration, it may be possible to precisely manage the number of Gd^3+^ ions inserted in the matrix, aiming at optimizing the enhancement of the MRI signal.

Moreover, the proposed new synthetic method could allow an improvement of the interaction of Gd^3+^ with the water environment as Gd^3+^ ions would likely incorporate within the superficial layers of ZnHCF particles.

Two kinds of particles of ZnHCF material were synthesized. The first, r-ZnHCF [20], was composed of micrometric particles and was the first identified in the literature as a cathodic material for Zn-ions batteries, providing the initial inspiration for this project. This material was used for preliminary investigations, although the micrometric size was not suitable for medical purposes. S-ZnHCF [36], instead, was studied in greater depth due to its nanometric size, which made it more suitable for injections as a contrast agent. Future developments of this work will be dedicated to the evaluation of these compounds as MRI contrast agents.

## 2. Materials and Methods

### 2.1. Synthesis of Rhombohedral ZnHCF (r-ZnHCF)

**Figure 1:**
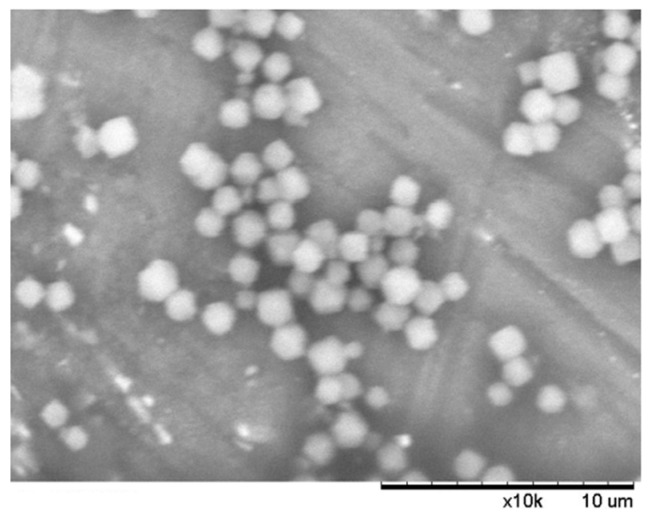
SEM image of r-ZnHCF.

r-ZnHCF was synthesized with a co-precipitation method found in the literature [20]. ZnSO_4_ H_2_O (2 mmol) was dissolved in 200 mL of ultrapure water, and K_3_Fe(CN)_6_ (2 mmol) was dissolved in 200 mL of ultrapure water. Then, the two solutions were mixed very quickly under vigorous stirring; the addition time was carefully controlled to obtain the correct shape of the particles, i.e., cuboctahedron-shaped ZnHCF. Then, the mixture was left under stirring for 24 h and then allowed to rest for 12 h. The resulting powder was filtered, washed with ultrapure water, and dried at room temperature. Then, the powder was heated at 70 °C to remove the coordinated water and transform the cubic phase into a rhombohedral structure. The obtained solid was characterized by SEM (Figure 1).

### 2.2. Synthesis of Spherical ZnHCF (s-ZnHCF)

**Figure 2:**
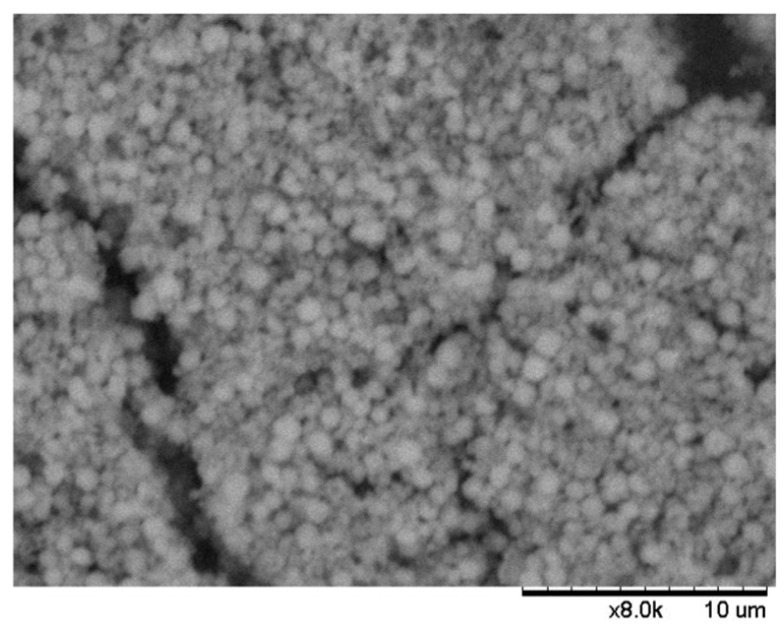
SEM image of s-ZnHCF, taken with the Hitachi TM-1000.

**Figure 3:**
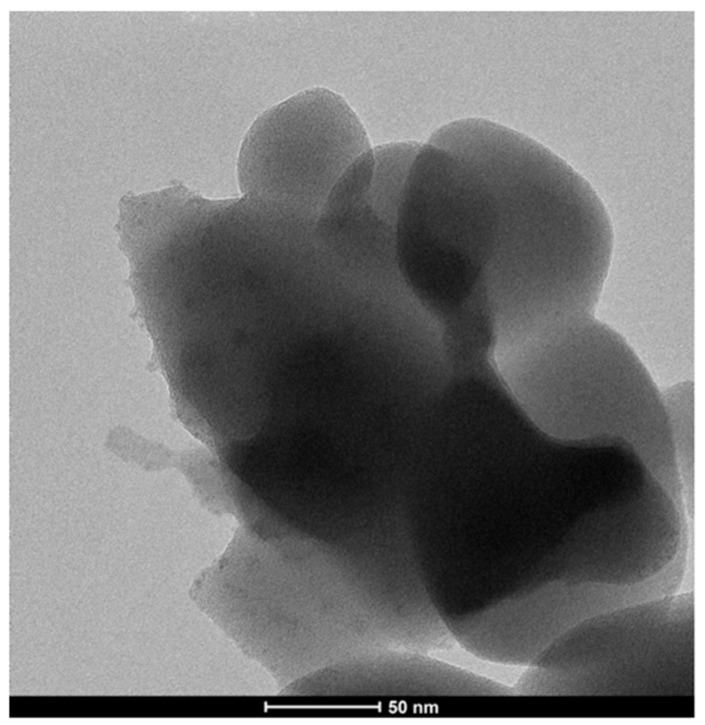
TEM image of s-ZnHCF.

**Figure 4:**
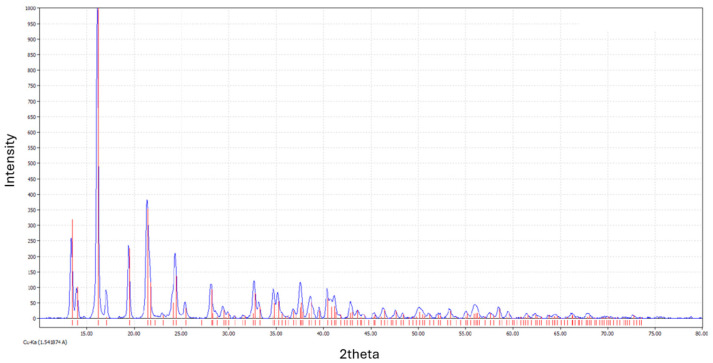
XRD diffractogram of synthetized s-ZnHC (blue line) and its theoretical pattern (red lines).

s-ZnHCF was synthesized as proposed in the literature [36]. Two solutions of 0.5 M K_3_Fe(CN)_6_ (80 mL) and 0.1 M ZnSO_4_ 0.1 M (80 mL) were simultaneously introduced in 50 mL of ultrapure H_2_O at 60 °C under vigorous stirring, by dropping them at the speed of 2 mL/min. Then, the suspension was allowed to settle for 3 h. The yellow precipitate was filtered, washed with deionized water, and dried at 70 °C overnight, after which it changed color to green. S-ZnHCF was characterized by SEM, TEM, and XRD (Figure 2, Figure 3 and Figure 4).

### 2.3. Preparation of the Working Electrode (WE)

FTO (fluorine-doped tin oxide)-coated glass was chosen as the electrical conductive support for the working electrode. An amount of 5 mg of r-ZnHCF or s-ZnHCF was suspended into 1 mL of ultrapure water with the help of the sonicator, obtaining a yellow suspension. A volume of 40 µL of the previous suspension (i.e., 0.2 mg or 3.2 mmol) was drop-casted on 1 cm^2^ FTO area, using a micropipette. This procedure was repeated 3 times to generate a uniform film on the FTO surface. Those two different working electrodes were named r-ZnHCF@FTO and s-ZnHCF@FTO. r-ZnHCF@FTO was finally dried at 70 °C overnight; while s-ZnHCF@FTO was left at room temperature for 5 days in air, following the changing of the color of the layer to green and then blue. The homogeneity of the layer was observed by stereomicroscopy.

### 2.4. Cyclic Voltammetries

Cyclic voltammetries (CVs) were carried out using a Palm-Sens Emstat4s HR potentiostat, controlled by PSTrace 5 software, in a 3-electrode cell: FTO with the relevant powder (both r- and s-ZnHCF nanoparticles were tested) as the working electrode (WE), Ag/AgCl with 3M KCl solution as the reference electrode (RE), and platinum foil as the counter electrode (CE). A 0.1 M gadolinium acetate (Gd(CH_3_COO)_3_) aqueous solution, degassed with N_2_ for 5–10 min, was used as the electrolyte, ranging in potential from 0 V to 1 V vs. RE, with a scan rate of 100 mV·s^−1^. r-ZnHCF@FTO was studied also in aqueous 1 M ZnSO_4_ (between 0 and 1.2 V vs. RE at a scan rate of 100 mV·s^−1^), and s-ZnHCF@FTO was characterized in both aqueous 0.1 M ZnSO_4_ and 0.1 M K_3_Fe(CN)_6_, from −0.2 to 1.1 V at 50 mV s^−1^.

### 2.5. Chronoamperometries

Chronoamperometries were performed using the same experimental setup used for CVs on s-ZnHCF@FTO in 0.1 M gadolinium acetate water solution, applying a potential to allow the incorporation of the powder with Gd. Then, the powder was analyzed with the energy-dispersive X-ray spectroscopy (EDX) technique to verify the presence of Gd^3+^ into the powder.

## 3. Results

The synthetic route to produce the two powders led to obtaining 316 mg of r-ZnHCF, with a yield of 77%, and 1.240 g of s-ZnHCF, with a yield of 98%. In both cases, the particles were formed by mixing two reagents, following the path reported in reaction 1:

(1) $$2K_{3}\left[{\mathrm{Fe}\left(\mathrm{CN}\right)}_{6}\right]+3{\mathrm{ZnSO}}_{4}\to {\mathrm{Zn}}_{3}{\left[\mathrm{Fe}{\left(\mathrm{CN}\right)}_{6}\right]}_{2}+3K_{2}{\mathrm{SO}}_{4}$$

SEM images (Figure 1) confirmed that r-ZnHCF consisted of crystals of a cuboctahedral shape and a rhombohedral structure, with a particle size diameter of about 2 µm, very similar to the SEM image found in the literature [20].

Regarding the characterization of s-ZnHCF, this powder was confirmed to be formed by nanoparticles of Zn_3_[Fe(CN_6_)_2_] with a diameter of about 50 nm (Figure 2 and Figure 3).

**Figure 5:**
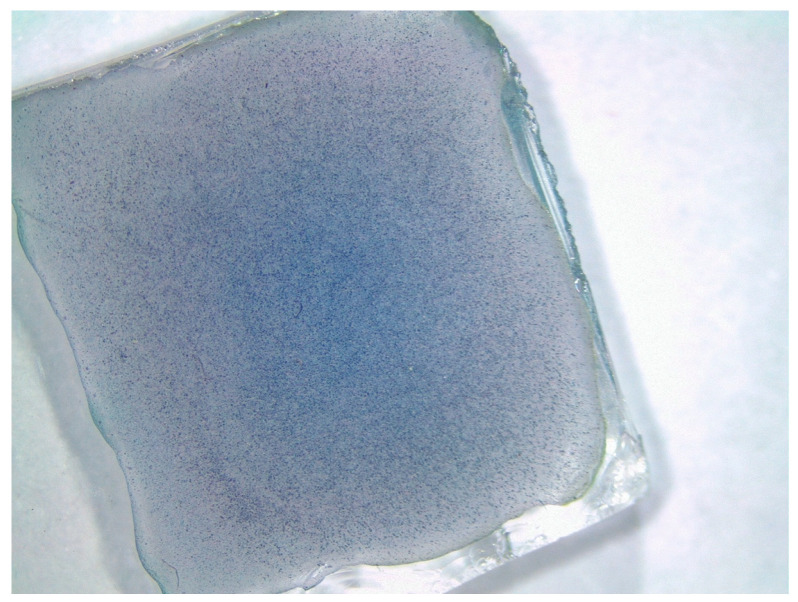
s-ZnHCF deposited onto the FTO surface.

The deposition of both powders onto the FTO surface using the drop-casting technique produced a uniform layer, as confirmed by stereomicroscopy. Figure 5 illustrates the homogeneity of the s-ZnHCF layer.

**Figure 6:**
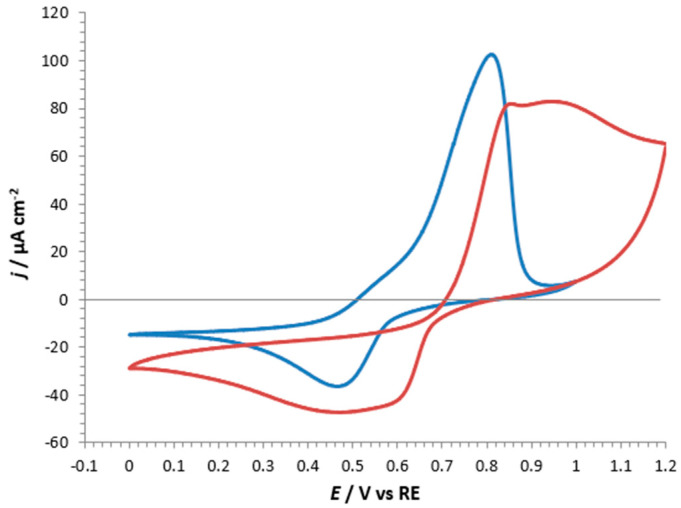
CVs on r-ZnHCF@FTO in 1 M ZnSO_4_ (red curve) and 0.1 M Gd(CH_3_COO)_3_ (blue curve). Scan rate 100 mV·s^−1^.

CVs were performed to investigate the cation incorporation capabilities, evidencing the different potentials for the processes carried out with different cations. Figure 6 shows CVs of incorporation and de-incorporation processes on r-ZnHCF@FTO in the presence of two different cations: Zn^2+^ and Gd^3+^. The test with zinc sulphate was performed to verify our experimental setup, comparing our results with those in the literature [20], which described r-ZnHCF as a promising material for cathodes in Zn-ion batteries, due to the possibility of a reversible Zn^2+^ cation incorporation process (see the red curve in Figure 6). Oxidation and reduction peaks, for the incorporation/de-incorporation reactions, were observed for both cations (see Figure 6), confirming the possibility of using ZnHCF to incorporate Gd^3+^ due to the similar ionic radii of Zn^2+^ (74 pm) and Gd^3+^ (94 pm).

In the case of Zn^2+^, CV showed two oxidation and two reduction peaks, the most intense of which fell at about 0.95 V (ox) and 0.6 V (red), respectively. When Gd^3+^ was investigated, only one oxidation and one reduction peak were visible, at about 0.8 V and 0.5 V, respectively. These peaks, for both the cations, corresponded to changes in the oxidation state of iron atoms, which must switch between +2 and +3 and vice versa when cation leaves or enters the powder, respectively. In the case of Zn^2+^, the incorporation/de-incorporation processes occurred at two slightly different potentials because Zn^2+^ could occupy two different lattice positions inside the hosting powder. More in general, the differences between the red and blue curves were due to the different chemical environments surrounding the two cations. The proposed mechanism for the Gd^3+^ incorporation is reported in reaction 2; the addition of one mole of Gd^3+^ in the system implied the expulsion of one mol of Zn^2+^ and the reduction of one of the two Fe^3+^ ions to Fe^2+^. Notably, the incorporation of one mole of Gd^3+^ implied the exchange of 1 mol of electrons (one Faraday).

(2) $${Gd}^{3+}+e^{-}+{{Zn}_{3}[Fe{\left(CN\right)}_{6}]}_{2}\to {Gd{Zn}_{2}[Fe{\left(CN\right)}_{6}]}_{2}+{Zn}^{2+}$$

**Figure 7:**
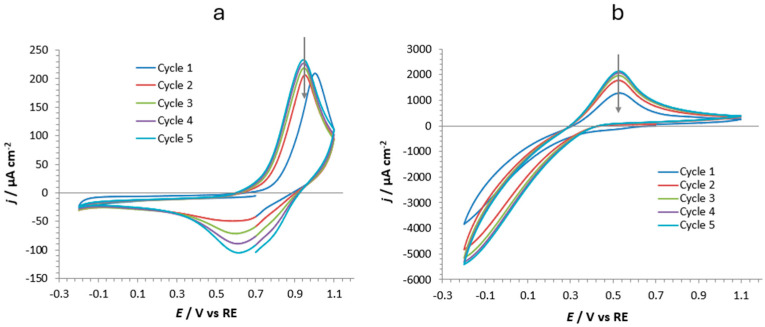
CVs (five cycles) of s-ZnHCF@FTO powder in (**a**) 0.1 M ZnSO_4_ and (**b**) 0.1 M K_3_Fe(CN)_6_ at 50 mV·s^−1^. The arrows indicate the decrease of peak current by increasing the number of cycles.

The same incorporation processes were carried out also on nanostructured powder, s-ZnHCF@FTO, to study the behavior of this hosting matrix when three different cations were exchanged: Zn^2+^, Gd^3+^, and K^+^. Potassium cation was chosen to investigate the effect of an ion with a significantly larger radius (152 ppm) than the other two, when entering a crystal system involving nanometric spheres (see Figure 2). Figure 7 shows the CVs of the incorporation processes for Zn^2+^ (a) and K^+^ (b) in the s-ZnHCF@FTO matrix. In the case of Zn^2+^, two peaks (one for oxidation and one for reduction) were clearly visible at 0.95 and 0.6 V vs. Ag/AgCl 3M, confirming the results obtained for the other type of hosting powder: r-ZnHCF@FTO. The differences between the two CVs’ shapes could be attributed to the dimension of the particles forming the deposited powder (compare the red curve in Figure 6 with the curves in Figure 7a) that can also affect the peak currents. On the contrary, the powder could not accommodate the large K^+^ cation inside its structure; the CVs were highly irreversible and distorted, without a clear reduction peak connected with the incorporation step.

**Figure 8:**
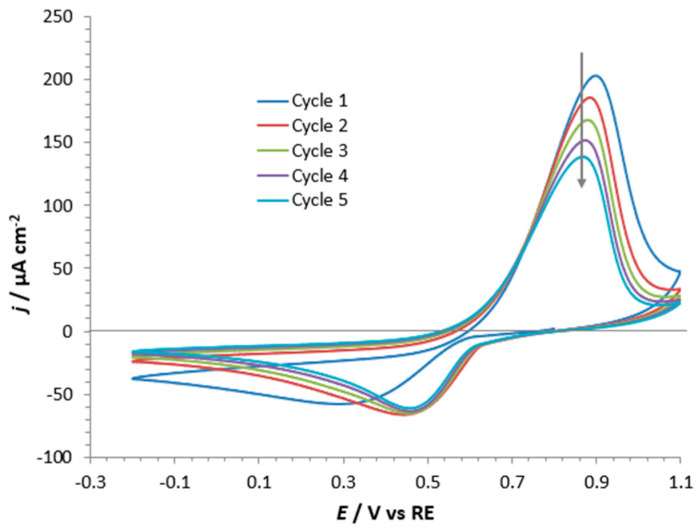
CV (five cycles) of s-ZnHCF@FTO powder in 0.1 M Gd(CH_3_COO)_3_ at 50 mV·s^−1^. The arrows indicate the decrease of peak current by increasing the number of cycles.

The ability of Gd^3+^ to incorporate into s-ZnHCF was confirmed by the presence of two visible oxidation and reduction peaks in CVs shown in Figure 8, at about 0.9 and 0.5 V, respectively. It is noteworthy that the reduction peak maintained a constant current intensity, while the oxidation peak decreased with cycles. This observation may serve as preliminary evidence for the stability of Gd^3+^ in the s-ZnHCF hosting matrix because, with time, Gd^3+^ substituted the Zn^2+^ already present in the crystal lattice and did not exit.

**Figure 9:**
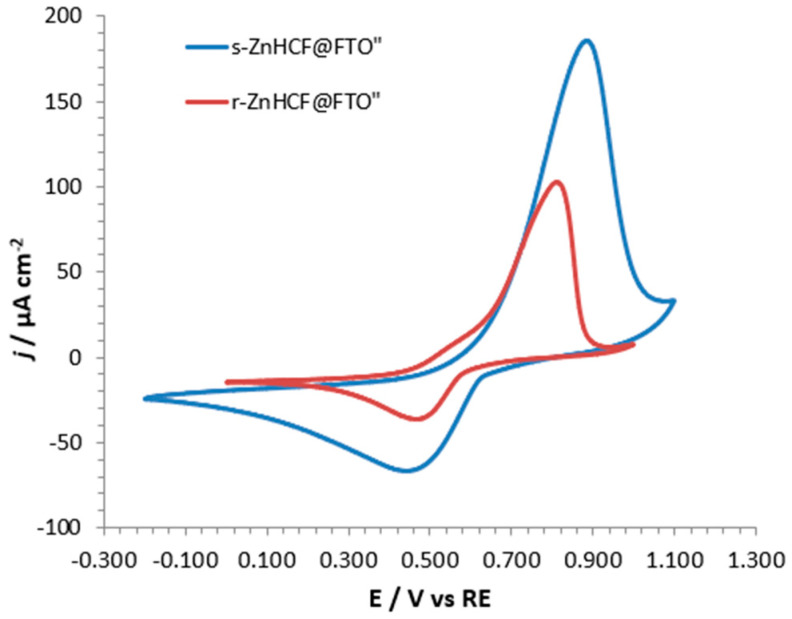
Overlap of the second CV cycle of r-ZnHCF@FTO (red curve) and of s-ZnHCF@FTO (blue curve) in 0.1 M Gd(CH_3_COO)_3_.

The shape of the CV curves obtained for Gd^3+^ incorporating s- and r- ZnHCF@FTO were very similar, only the peak current for s- was higher (see Figure 9). Very likely, the increased current was connected with the dimension of the particles; in fact, s-ZnHCF nanoparticles exposed a higher surface to the electrolytic solution with respect to r-ZnHCF micrometric particles.

**Figure 10:**
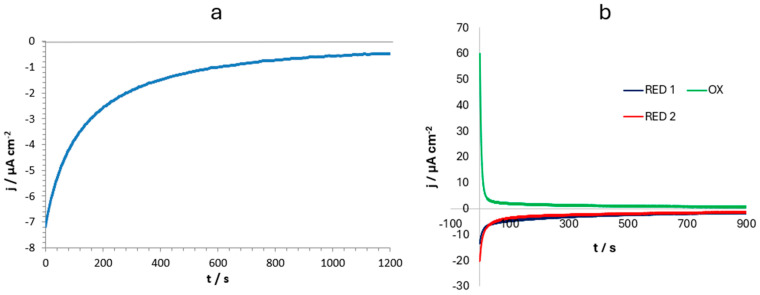
Chronoamperometry on s-ZnHCF@FTO to incorporate and de-incorporate Gd^3+^ in the hosting matrix. (**a**) Only one charge at 0.3 V vs. RE. (**b**) Two charge (at 0.3 V vs. RE) and one discharge (at 0.9 V vs. RE) processes. WE was dipped in 0.1 M Gd(CH_3_COO)_3_.

Having demonstrated the possibility of incorporating Gd^3+^ into both micro- and nanocrystalline ZnHCF powder, chronoamperometry experiments were carried out to charge and discharge the hosting powder with the selected cation. Figure 10a shows the cathodic decreasing incorporation current when a potential of 0.3 V vs. RE was applied for 20 min. By integrating the current, the amount of Gd inserted into the hosting powder was calculated, resulting in 1.93 × 10^−8^ mol, leading to a concentration of 9.65 × 10^−5^ mol·g^−1^ of hosting powder. To achieve a more precise determination of the gadolinium ion amount incorporated in ZnHCF, cavity microelectrodes (C-MEs) could be employed as they allowed for the investigation of a precisely known quantity of material [37].

To verify the stability and reproducibility of the Gd^3+^ incorporation process, a new set of chronoamperometries were performed. Figure 10b shows a reductive (incorporation) chronoamperometry performed at 0.3 V vs. RE for 15 min, followed by an oxidative (de-incorporation) step at 0.9 V vs. RE for 15 min and the repetition of the reductive process at 0.3 V for an additional 15 min, using a 0.1 M Gd(CH_3_COO)_3_ water solution. The test was carried out to investigate the charge transfer during the second incorporation process, after (partial) de-incorporation of the gadolinium ions, in order to investigate the stability of the powder. By integrating the current of the first reductive step (RED 1 in Figure 10b) up to 900 s, a total amount of Gd^3+^ was calculated, obtaining a value of 1.76 × 10^−8^ mol. This result was consistent with the Gd^3+^ amount obtained from Figure 10a, confirming the reproducibility of the experiments.

Considering the sum of RED 1 and RED 2 integration charge minus the OX integration charge, a total amount of 3.67 × 10^−8^ mol of Gd was obtained, which corresponded, after the three steps, to a concentration of 1.83 × 10^−4^ mol·g^−1^ of hosting powder.

Notably, with only two cycles, the Gd^3+^ content hosted by the powder was almost doubled, thus evidencing that not all Gd^3+^ was removed during the de-incorporation process.

Finally, in all the cases, the resulting current rapidly decreased and asymptotically approached zero, indicating that the matrix vacant sites were progressively filled, with a more rapid filling occurring during the first 5 min of the reductive potential application.

To confirm the presence of Gd atoms inside the matrix, EDX was employed to analyze the s-ZnHCF@FTO immediately after the chronoamperometry, after a surface washing procedure with ultrapure water.

**Figure 11:**
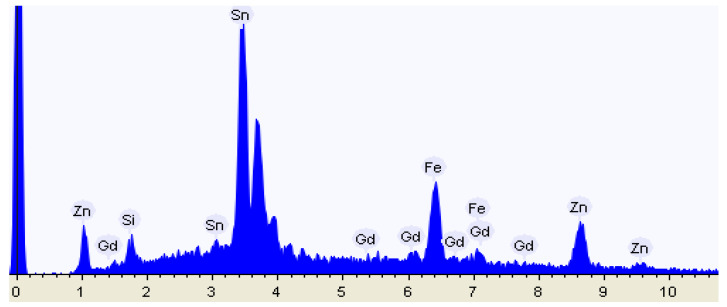
EDX spectrum of test 1.

Figure 11 displays the EDX spectrum after 20 min of incorporation potential (Figure 10a). As expected, the detected elements included Zn and Fe from ZnHCF and Sn from FTO. Traces of Si were also observed in the sample. Finally, Gd was detected by EDX, thus demonstrating that gadolinium was successfully incorporated into the structure of ZnHCF matrix.

Table 1 presents the weight percentages of Fe, Zn, and Gd, the components of the new gadolinium-rich zinc hexacyanoferrate. From the 9% of Gd detected after the run of Figure 10a (sample 1) and considering the ZnHCF amount deposited on the FTO surface, 1.14 × 10^−7^ mol of Gd could be calculated from EDX analysis. Since EDX is not a strictly quantitative technique, we can safely consider this value as compatible with the amount obtained from current integration.

After the three runs of Figure 10b, sample 2 contained a higher percentage of detected gadolinium (13%), which may be due to the succession of the reductive and oxidative steps, leading to a total amount of 1.66 × 10^−7^ moles. Finally, it is noticeable that the amount of Zn decreased significantly in sample 2, evidencing the substitution of Zn with Gd by cycling the electrode.

## 4. Discussion

Prussian blue analogues are promising materials in the medical field due to their biocompatibility and their ability to reversibly or irreversibly incorporate various metals. In this context, zinc hexacyanoferrate has been investigated as a candidate for creating GBCAs for MRI. EDX analysis has shown the possibility to introduce Gd^3+^ ions into its structure, which is the cation able to enhance the relaxivity of water, leading to more contrast-rich images in the traditional MRI. We proved the possibility of using a different cage, Prussian blue analogue, to contain the active atom.

Among the two powders presented in this study, s-ZnHCF is undoubtedly the preferred choice due to its nanometric size, which is more suitable for potential injection compared with the micrometric size, although an even smaller nanometric size would be ideal, leading also to a structural stable powder.

To facilitate their potential medical application, the powder was characterized as bare material deposited onto an FTO surface, without any additives or gluing agents. Only particular attention was paid to the time elapsed between the powder preparation and the incorporation test. In fact, a previous investigation demonstrated that an aging procedure allowed for a better adhesion of the powder to the FTO support. Thus, the powder was rested for 5 days before its usage. During this resting period, the powder changed color from yellow to green and then blue. FTO is an n-type wide band gap semiconductor, and FTO-coated glass is widely used for its optical transparency in the visible region, which allows light to pass through, along with excellent electrical conductivity [38]; this feature made it the support of choice for this project.

Chronoamperometry, conducted after cyclic voltammetry to identify incorporation potentials, allowed the insertion of about 10^−7^ or 10^−8^ moles of gadolinium in 0.2 mg of spherical nanoparticles of zinc hexacyanoferrate, as confirmed by EDX measurements. Specifically, 9% in weight of gadolinium was found in sample 1 and 13% from sample 2; the increase was attributed to the repetition of incorporation/de-incorporation processes (Figure 10b).

Thus, an electrochemical method was established for incorporating Gd^3+^, an ion with exceptional properties for enhancing visibility in magnetic resonance imaging, into a substrate with biocompatibility features. In a short time frame and with a simple setup comprising three electrodes immersed in a gadolinium electrolyte, it was possible to produce gadolinium-rich nanoparticles without additives or contaminants, solely through the application of a voltage.

It is important to note that this research is in its early stages, and further studies are necessary to optimize the amount of gadolinium carried by the ZnHCF matrix. This will involve investigating the correlation between the duration of the chronoamperometry, the applied voltage, and other experimental parameters with the extent of the incorporation process.

Ultimately, the feasibility of the system as an effective contrast agent will be evaluated by verifying, with inductively coupled plasma analysis, whether the modified nanoparticles release toxic free Gd^3+^ in water and in a biological environment. Additionally, the relaxivity (the main parameter to describe the efficiency of a contrast agent for MRI) of the new GBCA-containing solutions will be analyzed to evaluate any signal enhancements.

## 5. Conclusions

MRI, or magnetic resonance imaging, is a medical imaging technique that uses magnetic fields and radio waves to create detailed images of internal organs and tissues. Gadolinium-based contrast agents are used to enhance image contrast, making areas of interest, such as tumors or inflammation, more visible. This helps doctors make more accurate diagnoses and plan appropriate treatments.

In this research, an electrochemical method was studied to prepare a new type of nanometric contrast agent for MRI. This approach involved the use of a constant reductive potential of 0.3 V, determined through cyclic voltammetry studies, to a working electrode formed by FTO-coated glass covered by nanospheres of zinc hexacyanoferrate. Using this method, 1.93 × 10^−8^ and 3.67 × 10^−8^ mol of Gd^3+^ were incorporated into the nanoparticles. EDX further confirmed the successful incorporation of the gadolinium ion, which accounted for 9–13% of the total weight of the compound. Further studies and characterizations will be needed to evaluate the performance of those modified materials as contrast agents for medical use.

## Figures and Tables

**Table 1:** Data from EDX analysis.

#	Fe (w/w%)	Zn (*w*/*w*%)	Gd (*w*/*w*%)
Sample 1	59	32	9
Sample 2	69	18	13

## Data Availability

The original contributions presented in this study are included in the article. Further inquiries can be directed to the corresponding author.
